# Improvement in cardio-metabolic health and immune signatures in old individuals using daily chores (*Salat*) as an intervention: A randomized crossover study in a little-studied population

**DOI:** 10.3389/fpubh.2022.1009055

**Published:** 2022-10-24

**Authors:** Iftikhar Alam, Riaz Ullah, Attaullah Jan, Bismillah Sehar, Atif Ali Khan Khalil, Huma Naqeeb, Essam A. Ali, Qazi Muhammad Farooq Wahab, Mahpara Safdar, Abid Ali, Muhammad Haidar Zaman, Falak Zeb

**Affiliations:** ^1^Department of Human Nutrition and Dietetics, Bacha Khan University Charsadda, Peshawar, Pakistan; ^2^Department of Pharmacognosy, College of Pharmacy, King Saud University, Riyadh, Saudi Arabia; ^3^Department of Health and Social Sciences, University of Bedfordshire, Luton, United Kingdom; ^4^Faculty of Pharmaceutical and Allied Health Sciences, Institute of Pharmacy, Lahore College for Women University, Lahore, Pakistan; ^5^Department of Clinical Nutrition, Shaukat Khanum Memorial Cancer Hospital and Research Center, Peshawar, Pakistan; ^6^Department of Pharmaceutical Chemistry, College of Pharmacy, King Saud University, Riyadh, Saudi Arabia; ^7^Department of ENT, Khyber Teaching Hospital (KTH), Peshawar, Pakistan; ^8^Department of Environmental Design, Health & Nutritional Sciences, Allama Iqbal Open University, Islamabad, Pakistan; ^9^Boro Park Center of Rehabilitation, New York, NY, United States; ^10^Nan Shi Fu Zhong, Nanjing Normal University, Nanjing, China; ^11^Research Institute for Medical and Health Sciences, University of Sharjah, Sharjah, United Arab Emirates

**Keywords:** quality of life, religious prayers, religious practices, nutrition, immunity

## Abstract

**Background:**

Decline in cardio-metabolic health, immunity, and physical activity is associated with old age. Old people also find it difficult to engage in structured exercise programs. Therefore, there is a need to investigate common daily chores as an alternative for exercise that may also help in maintaining cardio-metabolic and immune health.

**Objective:**

We aimed to investigate whether *Salat*, an obligatory Islamic prayer involving various physical movements and closely resembling yoga, enhances the benefits conferred by the current guidelines for physical activity.

**Methods:**

A total of 30 overweight adults (mean (SD) age of 53.5 (8.7) years) participated in this study. For a 4-week duration, we compared the effects of *Salat* before/after meals (Pre-MS/Post-MS) on selected immunological and metabolic parameters in serum samples. We also compared the effects of both Pre-MS/Post-MS regimens in young and old subjects to observe any age-related effects.

**Results:**

Most of the baseline metabolic parameters and the count of immune cells were normal. Post-MS resulted in a significant reduction in body weight and percent body fat (%BF). Overall, Post-MS resulted in a clear leukocytosis with a significant increase in granulocytes, monocytes, and lymphocytes. When analyzing the lymphocyte compartment, a clear numerical increase was noted for T, B, and NK cells. The number of CD8+ T cells showed a statistically significant increase. Similarly, Post-MS induced leukocytosis in both young and old individuals, while the increase in granulocytes, monocytes, and lymphocytes was statistically significant in old subjects only.

**Conclusion:**

This study demonstrated that the Islamic obligatory and congressional *Salat* practice is capable of mimicking desirable pro-immune and pro-metabolic health effects.

**Clinical trial registration:**

(UMIN000048901).

## Introduction

The beneficial effects of physical activity (PA) in improving the overall aspects of health regardless of age are well documented (1, 2). Physical activity helps in the maintenance of general wellbeing (2), and in this way, it may help in reducing the age-related adverse effects and improving the overall quality of life (3). PA provides protection against non-communicable diseases (4). On the contrary, lower PA has been associated with a number of diseases, thus costing significantly as high as 1.5–3% of direct healthcare costs (5). World Health Organization (WHO) considers insufficient PA as the fourth leading risk factor for global mortality. Thus, to increase the amount of PA in the general population, especially in self-declared healthy older adults or those with specific chronic diseases, the WHO published new 2020 guidelines on PA (4) and recommends engaging in 150–300 min per week of moderate-intensity aerobic PA or 75–150 min per week of vigorous-intensity aerobic PA on at least 2 days in a week. Older adults are also advised to perform functional balance and strength training at moderate or greater intensity for at least 3 days per week.

With aging, there is a clear decrease in physical activity. As people get older, they are less interested in improving their health but more interested in retaining the health and capacities they already possess (6). Overall, the lack of a supportive community and acceptable social opportunities have been reported to be the main social obstacles to structural physical activity in old age (7, 8), in addition to social restrictions imposed by the COVID-19 pandemic. In addition, a sedentary lifestyle prevails more in less affluent societies, like those of most countries in the Islamic world (9). Given the circumstances, encouraging old people into doing some daily life activities or household chores as an interventional approach and as an alternative for structured physical activity and exercise has recently attracted the attention of many researchers (10–12). Similarly, it is known that activities that give a reason or opportunity for social interaction are perceived as less burdensome (10).

The pandemic of coronavirus disease 2019 (COVID-19) has pushed people apart from each other for their own safety. Preventive measures, such as limiting participation in several activities, including sports, exercise, and PA outside the home, have been recommended by various healthcare authorities throughout the world. These limitations have led to an increase in sedentary behavior and reduced levels of PA, which has further increased the risk of chronic health conditions in older adults. In this context, PA carried out in the home setting is recommended as an effective measure to stay healthy, boost immune system functioning, and improve general physical functioning and physical fitness. Home-based PA programs represent a potentially effective strategy for reducing sedentary behaviors, social isolation/distancing health consequences, and promoting enjoyable forms of PA during the COVID-19 pandemic. In such prevailing circumstances of lesser social life and in an effort to encourage sedentary individuals to engage in at least some physical activity, emphasis has shifted from promoting structured forms of exercise or structured physical activity to ordinary daily life activities or household chores, which can form our daily routine (13).

The activities of everyday life are considered to be enough to be physically active. However, the maximum physiological benefit can be achieved from such common daily life activities or household chores if these are done on a regular basis. Our interest was, therefore, to identify certain common daily life activities or household chores that may be espoused as part of physical activity for older persons (10) with a potential capacity for immunity and metabolic health improvement. We were of the view that such activities must comply with the sociocultural chucks of the study area, with enough motivation to engage the people (11), with some goal attainment scaling (12), and with the ability to provide an opportunity to socialize with others (9). One option may be *Salat*, which is an obligatory Islamic prayer, involving numerous physical movements and hence with a high potential to be adapted as a physical activity for old people. *Salat* is offered daily in a congressional manner with optimal opportunity for socialization and without any need for making special arrangements to perform it. Most importantly, *Salat* is highly adapted in the sociocultural context, and old individuals can easily engage in doing it on a regular basis with the highest degree of compliance. From the physical activity point of view, it is similar to other aerobic exercises, such as tai chi and yoga, and as such may improve physical fitness.

For the present study, we were mainly interested to investigate the effects of minor adjustments in terms of timing of physical activity (*Salat*) on certain metabolic and immune markers. We selected these health domains partly because of our extensive expertise in these areas and partly due to the findings of the age-associated compromised immunological status of old individuals in this locality. Despite a huge volume of data on the health benefits of post-prandial walking, no data were available on the health benefits of performing *Salat* before or after taking the meals (14–17). Based on extensive research literature (e.g., 15–17), we hypothesize that *Salat*, when considered as a low-to-moderate form of physical activity, may provide better physiological results if performed after the meals. We, therefore, aimed to determine whether prescribed *Salat* after meals confers long-term benefits comparable to walking after meals on a single occasion at any time of the day.

## Materials and methods

### Study population and sample

**Figure 1:**
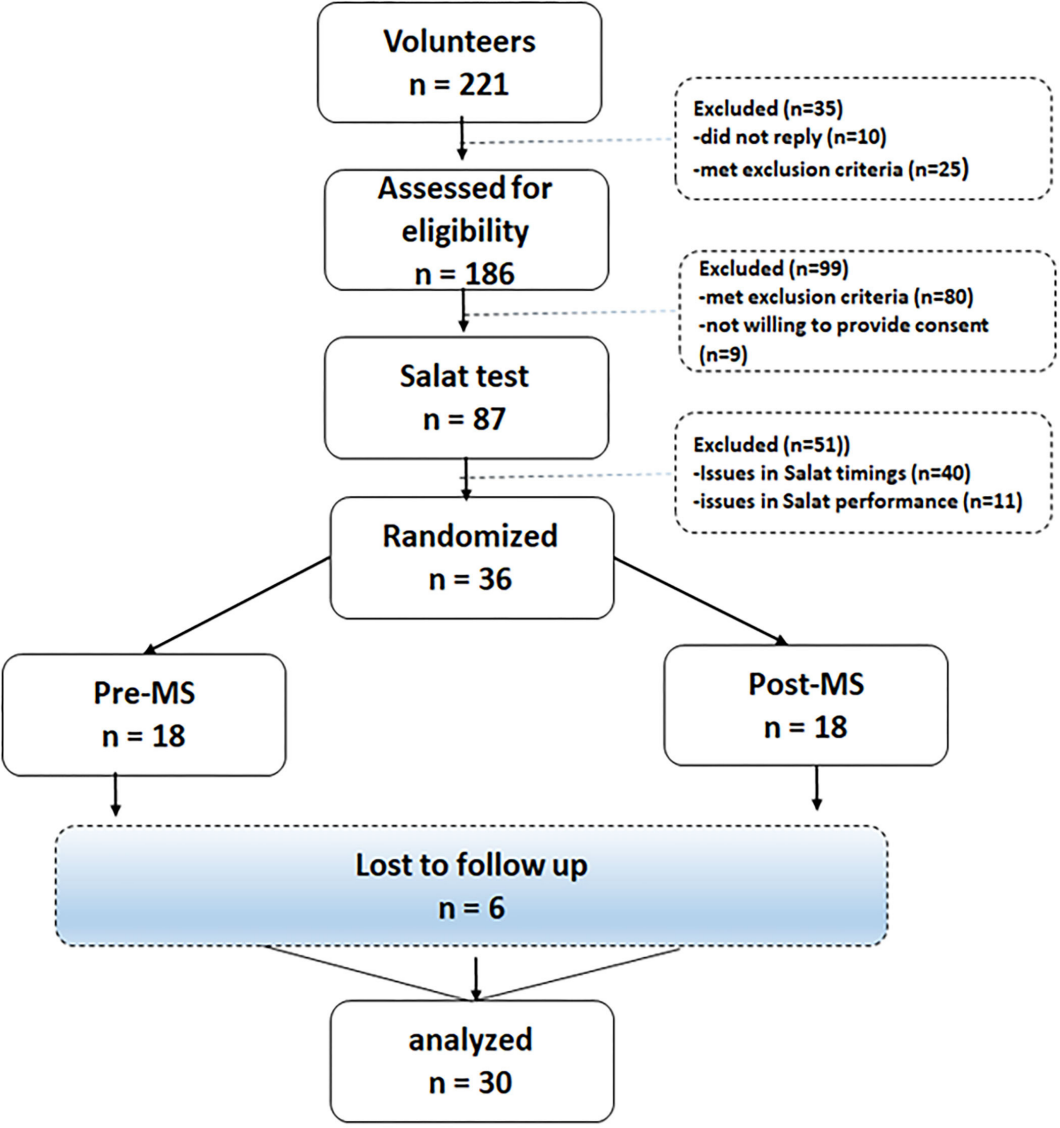
Participant's flow diagram.

In the mid of 2021, an internal advertisement was placed at the Nutrition Clinics of Nutrition Education, Awareness and Training (NEAT), a government-registered organization in Pakistan working for nutrition. The advertisement requested the NEAT members and/or their relatives to participate in the study. Interested volunteers [*n* = 221] were sent a document in the local languages (Pashto and Urdu) with all relevant information regarding study procedures. The selected respondents [*n* = 186] were invited for an online eligibility interview. The respondents [*n* = 87] were considered eligible if they fulfilled the eligibility criteria. Eligible volunteers were invited to demonstrate a final *Salat* test to observe their overall *Salat* performance, level of intensity, the structure of *Salat*, and the time taken. On the completion of the *Salat* test, 36 individuals were selected for the final study. However, six people dropped out at various stages of the study, and the final sample included 30 participants. The sample size (18) is consistent with published recommendations for evaluating the effect of exercise (before or after meals) on various health outcomes (19, 20). Figure 1 provides a schematic overview of the participant selection flow.

### Study settings

The study was conducted from December 2021 to February 2022 at the NEAT nutrition clinics. The current post-meal *Salat* crossover study involved two interventions in a randomized manner, separated by a 2-week washout period. Within each 4-week intervention, participants attended the clinics of NEAT on days 1, 15, and 30. On all these days (three time points), data were collected on the parameters like anthropometrics, 24-h dietary recall (24-HR DR), BP, habitual physical activity of the previous 2 weeks, and fasting blood samples.

### Ethical approval and trial registration

The Research Ethics Committee of NEAT approved the study (NEAT/R.21/2021), and participants provided written consent before commencing the study. The study was registered under clinical trial No. UMIN000048901.

### Participants and eligibility criteria

A total of 30 medically fit but overweight (BMI: 26-29) participants aged 40–80 years, who are non-hypertensive, and who are non-diabetic were recruited. The other eligibility criteria were being non-smokers, non-exercisers, and regular Salat performers. Participants with any physical comorbidity that would have required them to offer *Salat* in a sitting position only or on a chair were excluded. A consultant endocrinologist monitored the participants included in the study, but this did not preclude participants from visiting their usual healthcare provider if required.

### Randomization and masking

Randomization protocol was generated by computer and determined the intervention order before the study began. A biostatistician helped in undertaking the protocol and was accessed only after the participants were enrolled in the study.

### Nature of intervention

*Salat*, as a common religious prayer offered five times a day in a congressional manner, was used as an intervention. The only modification done was to ask the participants to take their meals either before or after they perform their daily *Salat* prayers. The participants were asked to adhere to their daily *Salat* patterns without any other changes in terms of time, intensity, or related patterns of *Salat*. We did not measure the equivalent exercise intensity of *Salat*; however, based on the previous reports (18, 21–23), *Salat* was found to be equivalent to “moderate” physical activity in terms of intensity.

### Study protocol

To comply with the current physical activity guidelines, both interventions required participants to offer *Salat* each day. The “pre-meal *Salat*' intervention (Pre-MS)” involved offering at least two *Salat* sessions before the two major meals, i.e., the afternoon *Salat* before lunch and the late night *Salat* before dinner. The “post-meal *Salat* intervention (Post-MS)” asked participants to offer *Salat* after each of these two main meals, starting within 5–10 min of completing the meal. We asked the participants to comply with these protocols during two out of the five *Salat* sessions in a day, i.e., the noon *Salat* (*Duhur:* after 12–14 h) and the night *Salat* (*Ishaa:* 60–90 min after sunset). These two time points of *Salat* were purposely selected as, usually, these *Salat* times coincide with eating the meals, lunch and dinner, respectively. The other three *Salat* sessions were not included as part of the protocol. The morning breakfast was not included, as the early morning *Salat* (*Fajar:* 4:30–5 h) is performed very early in the morning before dawn, and we felt that it was not possible and also feasible to comply with the current protocol for the early morning *Salat*. Similarly, the timings of late afternoon *Salat* (*Assar:* 15–16 h) and late evening *Salat* (*Maghreb:* just after the sunset) were not the usual eating times in the study area and therefore were excluded in the present protocol. Participants were advised not to change their diet or lifestyle habits during the study period, beyond complying with the prescribed *Salat* regimen.

In terms of the time required for offering *Salat* five times a day, we did some small piloting trials before the start of the actual study. We interviewed adult Muslims who performed *Salat* regularly and asked them about the time they took for performing *Salat*. In addition, we also visited various mosques and noted the actual time required to complete one full cycle of *Salat*. Based on interview information and our own observations, we asked the participants to complete their *Salat* within a time frame as given in the following: *Fajar Salat* (10–20 min), *Duhur Salat* (10–15 min), *Asar Salat* (10–15 min), *Maghreb Salat* (10–15 min), and *Ishaa Salat* (15–20 min).

### Socio-demographics and anthropometric measurements

Sociodemographics include self-reported information on age, income sources, etc. Anthropometric measurements include weight, height, and BMI and were determined as reported previously by Alam et al. (24). Briefly, weight was measured with respondents wearing light clothes, being bare feet, and leaving on loose hair and without any type of accessory on their heads. The digital scale used to measure weight was the TANITA Weight Scale (HD-662 Digital Weight Model–Black Japan), with a capacity of 150 kg and an accuracy of 100 g. The weighing was performed by asking the interviewee to place their feet on the center of the scale platform and remain in an upright position, with arms stretched along the body and without moving. The reading and recording (kg) were performed immediately after the weight value was fixed on the display, without oscillations. Height was measured using a non-stretchable measuring tape. Two measurements of weight and height were taken, and the mean values were considered for further calculations and analysis. BMI was calculated using weight and height measurements using the formula (BMI = Weight (Kg) divided by height (cm squared).

### Blood pressure determination

Blood pressure was measured using a standard mercury sphygmomanometer (2 mmHg per unit). The subject was asked to sit relaxed in a chair with his arm supported comfortably, and the pressure cuff was applied close to the upper arm. The cuff was rapidly inflated to a pressure above the level at which the radial pulse could no longer be felt. The stethoscope was placed lightly over the brachial artery, and the mercury column was immediately allowed to fall at the rate of 2 mmHg per second. The first perception of the sound was taken as the systolic pressure, and then, the mercury was allowed to fall further till the sound ceased to be tapping in quality, became fully muffled, and finally disappeared. The level where it disappeared was taken as the diastolic pressure. The cuff was then deflated to zero pressure. Prior to measurement, the subjects avoided strenuous exercise. They were asked to be mentally relaxed and rested quietly for 5 min before measurement. Two BP measurements were taken 1–2 min apart and averaged for records. An additional measurement was required if the first two readings differed by > 5 mmHg, and the mean value of the three readings was recorded.

### Biochemical quantification

Blood collection was conducted by the field investigation team, and all subjects fasted for at least 8 h. Fasting glucose and blood lipids were analyzed on a Cobas C-311 analyzer with assay kits (Roche Diagnostics, Risch-Rotkreuz, Switzerland). The lipid panel test disk quantitatively determined TC, HDL-C, and TG and provided a calculated value for LDL-C using the Friedewald formula when the concentration of TG was < 4.52 mmol/L (LDL = TC–HDL–TG/5). Moreover, non-HDL-C was calculated as TC minus HDL-C. Instrument calibration was evaluated by checking the system using a solution, namely “control samples.” Blood glucose levels were measured according to the standard procedures described by the manufacturer's instructions.

For the assessment of immune signatures, whole blood samples were used. The samples were processed following a procedure on whole frozen blood and a special analytical procedure developed and validated by us (20). Frozen blood was thawed in a 37°C water bath, and red cells in the blood samples were lysed with saline and water. The cells were then stained with 50 μl of the antibody cocktail and incubated for 30 min at room temperature in the dark. The monoclonal antibodies and fluorescent conjugates used were CD3 (Pacific Orange), CD4, CD8, CD16, and CD19. Cell populations were measured by using a flow cytometer and the acquisition commercial software BD FACSDiva (Becton Dickinson).

### Data quality management

The present study was a community trial, where quality assurance is fairly challenging, particularly compliance to intervention. We managed this issue by adopting multiple strategies: (1) the participants of the present study were a sub-sample of our previous studies (24, 25), and the subjects we selected were familiar with such studies; (2) the protocol of *Salat* was conducted in a congressional manner in the same mosque and hence all the participants complied appropriately; (3) the participants we selected for the present study had a reputation as regular *Salat* performers; and (4) the participants marked their attendance in a notebook each time they performed *Salat* in the presence of the Imam of the Mosque. In addition, each subject was reached by cell phone during the experimental phase to stimulate compliance and record any adverse events. In addition, text messages were sent two times weekly to remind subjects to perform *Salat* as per the protocol. These messages began with the Islamic call for *Salat* (Adan). Throughout the study, participants kept a *Salat* performance diary, recording the beginning and end times of each *Salat*. This was used to calculate the time it took for the participants to perform *Salat*. Compliance was checked for every subject by verifying their performance diary on a weekly basis during the scheduled visits of the participants to the NEAT clinics. The compliance of the period between the first and second visits, between the second and third visits, between the third and fourth visits, and the global compliance mean of each period compliance were calculated and expressed as percentages. Per day compliance of lower than 80% was considered a deviation from the protocol.

### Statistical analysis

We used a convenience sample technique and recruited a cohort from the population of our previous studies on nutrition and immune functions in young and old age groups (24, 25). The sample we selected had been familiar with the research study of the same nature, and this was an important factor in sample selection to get the maximum possible compliance to the interventions. Data were entered, cleaned, and coded in Microsoft Excel. Analyses were undertaken using GraphPad Prism. A mixed model, which included a term for order, was used to analyze the data. The results are presented as differences (with 95% CIs) between the two interventions. The “matched pairs analysis” was used for the calculation of differences. Normally distributed parameters were compared using the Student's paired *t*-test, whereas non-normal distributed parameters were compared using the Wilcoxon signed-rank test. All results are shown as mean ± SD, unless otherwise stated. A *p* ≤ 0.05 was considered significant.

## Results

Table 1 shows the baseline characteristics of the study participants as a whole. Our preliminary analysis showed no significant differences in the baseline characteristics of the subjects (data not shown; p, for all trends >0.05). Participants in this study comprised young and old individuals. All participants were overweight (mean BMI = 28.6) with a high body fat percentage. Except for energy, intake of other nutrients was within normal range. The saturated fat content of the diet was high. Most of the baseline metabolic parameters were normal. Similarly, immune cells were mostly within the normal range.

**Table 1:** Baseline characteristics of the study participants (*n* = 30).

**Characteristics**	**Mean (SD)**
Age (years)	53.5 (8.7)
Weight (Kg)	83.4 (12.7)
BMI (Kg/m^2^)	28.6 (3.7)
%BF	29.4 (11.4)
Energy intake (Kcal/day)	2,843 (1,121.7)
Protein intake (g/day)	63.3 (9.6)
Fat intake (g/day)	89.2 (14.3)
Saturated Fat intake (%TE)	34.2 (12.6)
Fiber intake (g)	2.6 (2.1)
Systolic BP (mm of Hg)	147.1 (24.4)
Total cholesterol	5.4 (1.3)
HDL Cholesterol	1.9 (1.2)
Triglycerides	1.7 (0.3)
Leukocytes (10^9^/L)	5.1 (2.1)
Neutrophils (10^9^/L)	2.8 (1.3)
Eosinophils (10^9^/L)	0.4 (0.2)
Basophils (10^9^/L)	0.3 (0.2)
Monocytes (10^9^/L)	0.6 (0.1)
Lymphocytes (10^9^/L)	1.5 (0.7)
CD3+T cells (10^9^/L)	3.4 (1.3)
CD4+ T cells (10^9^/L)	0.5 (0.1)
CD8+ T cells (10^9^/L)	0.5 (0.1)
CD19+ B cells (10^9^/L)	0.6 (0.1)
CD16+ NK cell (10^9^/L)	0.7 (0.2)

BMI, body mass index; %BF, percent body fat; HDL, high-density lipoprotein; CD, cluster of differentiation.

**Figure 2:**
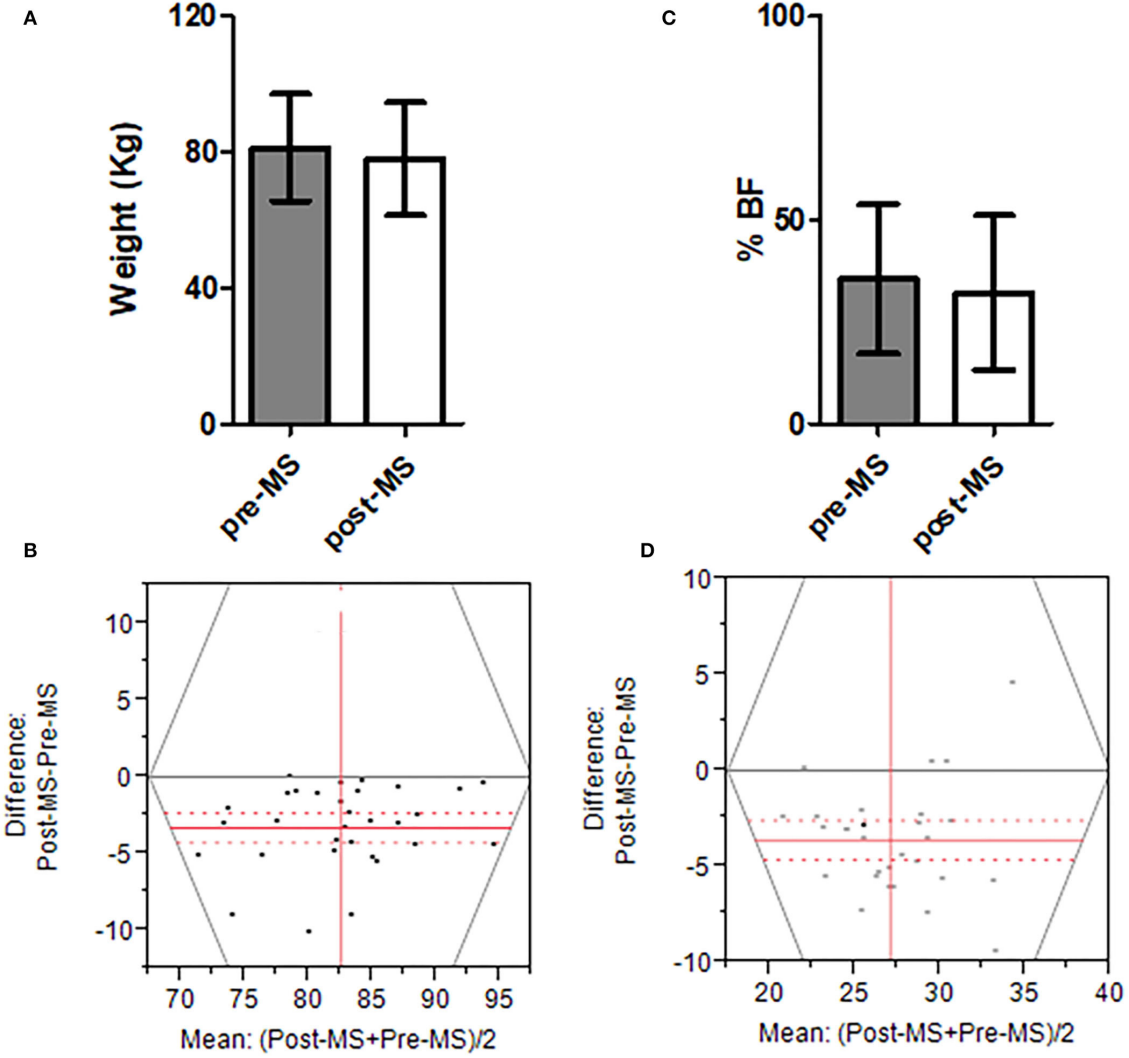
Mean body weight Pre- and Post-MS *Salat* regimens. **(A)** The mean body weight of Pre-MS and Post-MS regimens. **(B)** The difference in body weight between Pre-MS and Post-MS regimens. **(C)** %BF of Pre-MS and Post-MS regimens. **(D)** The difference in %BF between Pre-MS and Post-MS regimens. In **(B,D)**, the gray solid line shows “zero difference”. The red solid line is the mean difference. The dashed red lines are 95% confidence bands. The *P*-value for all analyses was < 0.05. All analyses were performed using “matched paired analyses”.

Figure 2 shows the mean body weight at pre-MS and post-MS *Salat* regimens (Figure 2A). Post-MS resulted in a significant reduction in body weight, the difference being −3.3 kg (CI: −4.27–−2.32; *p* < 0.0001) (Figure 2B). There was also a significant reduction in %BF by practicing post-MS *Salat* regimen (% Mean difference, −3.6344; 95% CI: −4.65–−2.60; *p* < 0.0001) (Figures 2C,D).

Table 2 shows mean (SD) measurements of immune cells and metabolic parameters with the Pre-MS *Salat* and Post-MS *Salat* regimens. Overall, Post-MS resulted in a clear leukocytosis with a clear numerical increase in the number of granulocytes, monocytes, and lymphocytes. When analyzing the lymphocyte compartment, a clear numerical increase was noted for T, B, and NK cells. Although CD8+ T cell numbers showed a statistically significant increase after *Salat*, the absolute increase was very limited. The mean CD4/CD8 ratio, an age-appropriate value, was not significantly increased. However, the levels of HDL cholesterol (*p* = 0.0343) and triglycerides (*p* = 0.0001) changed significantly.

**Table 2:** Comparison of immunological and metabolic parameters between Pre-MS and Post-MS regimens.

**Parameters**	**Pre-MS**	**Post-MS**	**Difference (95% CI) (Post-MS–Pre-MS)^a^**	*p*-value^*^
Leukocytes (10^9^/L)	4.8 (1.2)	5.5 (1.4)	0.7 (4.33–5.01)	0.0420
Neutrophils (10^9^/L)	2.6 (1.2)	3.1 (1.4)	0.5 (0.42–0.51)	0.1429
Eosinophils (10^9^/L)	0.3 (0.1)	0.6 (0.3)	0.3 (0.18, 0.42)	< 0.0001
Basophils (10^9^/L)	0.2 (0.2)	0.5 (0.3)	0.3 (0.16, 0.43)	< 0.0001
Monocytes (10^9^/L)	0.3 (0.2)	0.6 (0.3)	0.3 (0.63, 0.43)	0.001
Lymphocytes (10^9^/L)	2.7 (0.4)	4.4 (0.5)	0.3 (0.71, 0.53)	0.0129
CD3+T cells (10^9^/L)	0.8 (0.3)	1.4 (0.3)	0.3 (0.14, 0.46)	0.0003
CD4+ T cells (109/L)	0.5 (0.2)	0.8 (0.3)	0.3 (0.17–0.43)	< 0.0001
CD8+ T cells (109/L)	0.4 (0.3)	0.7 (0.3)	0.3 (0.14, 0.45)	0.0003
CD19+ B cells (109/L)	0.4 (0.2)	0.7 (0.3)	0.3 (0.17, 0.43)	< 0.0001
CD16+ NK cell (109/L)	0.5 (0.3)	0.8 (0.4)	0.2 (0.45, 0.46)	0.0124
Systolic BP (mm of Hg)	141.4 (26.1)	128.2 (27.2)	0.8 (−0.5, 5.2)	0.0001
Total cholesterol	5.4 (1.4)	5.2 (1.6)	−0.7 (−1.48, 0.08)	0.076)
HDL Cholesterol	2.2 (1.3)	1.5 (1.2)	−0.8 (−1.341, −0.05)	0.0343
Triglycerides	2.0 (0.4)	1.3 (0.8)	−0.7 (−1.03, 0.37)	0.0001

CD, cluster of differentiation, Pre-MS, Salat practice before the meals, Post-MS, Salat practice after the meals.

* Significant at p ≤ 0.05. **Significant at p ≤ 0.01.

Aging has pronounced effects on the immune system, as we also noted in our previous studies on this population (17, 19). We, therefore, separated the subjects into two distinct groups (young and old) in order to compare the effects of both Pre-MS and Post-MS on young (*n* = 16) and old subjects (*n* = 14). Although Post-MS-induced leukocytosis was seen in both young and old individuals, an increase in granulocytes, monocytes, and lymphocytes was statistically significant in older subjects but did not reach significance in young subjects (Table 3).

**Table 3:** Comparison of immunological and metabolic parameters between Pre-MS and Post-MS regimens in young and old individuals.

**Parameters**	**Young (*****n*** = **14)**		**Old (*****n*** = **16)**	
	**Pre-MS**	**Post-MS**	**Pre-MS**	**Post-MS**
Leukocytes (10^9^/L)	5.4 (1.3)	4.6 (1.4)	4.9 (1.3)	5.4 (1.4)^**^
Neutrophils (10^9^/L)	5.4 (1.2)	2.4 (1.3)*	2.9 (1.1)	3.4 (1.2)*
Eosinophils (10^9^/L)	0.4 (0.2)	0.5 (0.3)	0.3 (0,1)	0.6 (0.1)^*^
Basophils (10^9^/L)	0.4 (0.2)	0.7 (0.2)	0.3 (0.1)	0.6 (0.2)^**^
Monocytes (10^9^/L)	0.4 (0.3)	0.7 (0.4)	0.4 (0.1)	1.2 (0.2)
Lymphocytes (10^9^/L)	2.8 (1.5)	4.2 (1.6)	2.7 (1.4)	4,6 (1.1)
CD3+T cells (10^9^/L)	1.3 (0.3)	1.4 (0.4)	0.5 (0.3)	0.8 (0.6)^*^
CD4+ T cells (109/L)	0.6 (0.3)	0.9 (0.4)	0.4 (0.2)	1.2 (0.3)^*^
CD8+ T cells (109/L)	0.3 (0.1)	0.6 (0.3)	0.6 (0.1)	0.9 (0.2)^*^
CD19+ B cells (109/L)	0.3 (0.2)	0.7 (0.3)	0.4 (0.1)	0.8 (0.3)
CD16+ NK cell (109/L)	0.3 (0.2)	0.6 (0.3)	0.8 (0.2)	0.9 (0.3)
Systolic BP (mm of Hg)	131.2 (25.3)	119.5 (26.9)	149.5 (23.2)	136.8 (31.1)^*^
Total cholesterol	5.0 (1.5)	5.2 (0.2)	5.1(1.1)	5.2 (1.2)^*^
HDL Cholesterol	1.7 (1.1)	1.0 (1.6)	2.7 (1.1)	2.0 (0.2)^*^
Triglycerides	2.1 (0.6)	1.3 (0.2)	2.1 (0.6)	1.2 (0.3)^**^

CD, cluster of differentiation; Pre-MS, Salat practice before the meals, Post-MS, Salat practice after the meals.

* Significant at p ≤ 0.05.

** Significant at p ≤ 0.01.

## Discussion

Our present study had three main goals: first, we were interested to investigate whether *Salat* could serve the purpose of mild physical activity. Second, we were interested in determining whether the timings of offering *Salat* before or after the two main meals of the day showed any difference in affecting the selected metabolic parameters and immune signatures. Finally, we wanted to study the possible differential effects of pre- or post-meal *Salat* interventions separately on young and old individuals in an effort to see the effects of aging. With these goals, our study findings may be summarized as follows: first, the Islamic obligatory and congressional *Salat* practice is capable of mimicking desirable pro-immune and pro-metabolic health effects (Table 2). Second, the positive effects are more evident in the post-meal practice of prayers (Table 2). Finally, these effects are more profound in old individuals when compared to young individuals (Table 3). These findings have many important clinical implications and can be used while planning exercise regimens for old individuals, particularly for those who may face sociocultural obstacles in their daily life to practice planned physical activity or exercise.

*Salat* is a unique model of physical activity for old people, as it is a form of complete physical activity and meditation (26). Being obliged to pray at specific times of the day, higher compliance is usually observed. *Salat* involves recitations and specific body positions, for example, standing, bowing, prostration, sitting, and turning the head to the right and then to the left shoulder (27). Muslims are required to perform *Salat* five times daily in addition to voluntary prayers, thus providing an opportunity for small bouts of physical activity (28). As a form of exercise and meditation, it has a positive influence on physiological parameters, such as heart rate (27), blood pressure, and respiration rate (21). Therefore, *Salat* is similar to any other physical activity, and meditation can be used as a therapy for patients with various ailments (22).

As depicted by the images provided in Supplementary file 1, *Salat* involves the motion of the human body in a variety of directions and angles (23). In this way, it is similar and equivalent to *tai chi* yoga as investigated in a number of studies. Recent studies of yoga (18) and *Salat* (29) showed a decrease in systolic blood pressure when an individual performs yogic relaxation and meditation. Our study showed that *Salat* decreased systolic BP and positively affected other metabolic parameters and immune signatures, including HDL and triglycerides. This may reduce the sympathetic discharge, resulting in and predominance of the parasympathetic system (29). Ibrahim et al. (30) suggested that subjects who perform *Salat* regularly, five times a day, would have a healthy body composition, improved basal metabolic rate, and reduced body fat mass.

Our results also showed that Post-MS is more effective compared to Pre-MS regimen in inducing positive effects on the numerical value of immune cells and mean value of metabolic parameters. Previous studies on the positive effects of post-prandial exercise reported improvement in a number of metabolic health parameters (14–17). As an example, Reynolds et al. (16) conducted a randomized, crossover study on 41 adults with type 2 diabetes mellitus. They found significant improvement in the metabolic parameters of their patients in response to walking after they ate their meals. The real mechanism of how exercise after meals may induce positive effects on immune cells is still not clear. However, these positive effects on the numerical number of immune cells may be due to improvement in post-prandial glycemia induced by exercise (16, 31, 32). In addition, any loss in weight and body fat, as observed in the present study (Figure 2), has been shown to be effective in generating positive effects on the overall immune parameters (12, 13).

The current study also showed that old compared to young individuals displayed strong positive responses to Post-MS (Table 3). One possible reason for this observation may be that old age is associated with a longer past experience of *Salat*, and any change or intervention in such a regimen could be more profound in old individuals when compared to young individuals. This observation credits for future studies. In old age, extensive changes in the immune system occur. We previously demonstrated that old Pakistani individuals have a weaker immune system when compared to young (12, 13). Therefore, any intervention effort, similar to the one performed in the current study, to improve their immune health may be more effective in the old when compared to the young.

The present study has certain limitations. First, we could not include female participants due to cultural reasons (13). The inclusion of female subjects may give some more interesting insights, as they have even more physically restricted lifestyle patterns in this part of the world, but have the same obligation for performing *Salat* as the male subjects do. Future studies must consider the inclusion of female participants. Second, due to scarcity of resources, we could not standardize *Salat* in terms of exercise intensity and amount of energy burnt. While these are important aspects of *Salat* that need to be investigated to accurately know its physiological benefits in more detail, some previous investigations (18, 23, 29, 30) reported that *Salat* is equivalent to yoga in terms of intensity and many possible health benefits. Third, the sample size of 30 seems to be a small sample. However, given the nature of the study context, where there is no tradition of considering religious activities as an area of scientific research, the sample looks appropriate to maintain accuracy and compliance.

Based on our findings, we recommend that everyday life activities, like *Salat*, should gain more attention in health prevention programs, as it may be more practicable to motivate older people to (re)engage in the types of physical activity they feel more familiar with while realizing equally positive effects on health status. Interventional approaches may need to address potential motivators (e.g., religious obligations here), as well as barriers to daily life activities (10). Since household chores are usually considered obligations rather than pleasure, identifying motivators for engaging in these activities is crucial. Strategies that have been shown to encourage physical activity include motivational interviewing (11) and goal attainment scaling (12). Similarly, it is known that activities that give a reason or opportunity for social interaction are perceived as less burdensome (10). We anticipate that all these characteristics are found in *Salat* and hence recommend that old people, in particular, be motivated to engage in *Salat* in the prescribed manner. Finally, the types of religious prayer presented in this paper might give an impression of religious biasness on the part of the authors of the current report. Nevertheless, the close connection between religious practices and health is historically very old (33). A vast volume of literature encourages the combining of religious activity and exercise through “prayer walking” and “walking meditation” (31, 32). Any exercise protocol that has been proven to be beneficial to human health should be, therefore, considered beyond its religious aspect (34).

Given that *Salat* may be a good alternative to moderate physical activity (18, 23, 29), the older persons may spend 50-80 min in a day while offering *Salat*. This will be in compliance with the WHO's recommendations of moderate aerobic activity for older persons (4, 35, 36).

## Conclusion

Our present study demonstrated that the Islamic obligatory and congressional *Salat* practice is capable of mimicking desirable pro-immune and pro-metabolic health effects. Moreover, these effects are more evident in the post-MS group when compared to the Pre-MS group, an important finding that further necessitates the importance of fine-tuning the already existing daily life activities in a way to transform them to be more effective in inducing health benefits. In addition, these effects are more profound in old individuals when compared to young, an interesting finding that must encourage the researchers to further investigate other possible benefits of *Salat* or *Salat*-like physical activities for old individuals.

## Data availability statement

The original contributions presented in the study are included in the article/Supplementary material, further inquiries can be directed to the corresponding author.

## Ethics statement

The studies involving human participants were reviewed and approved by the Research Ethics Committee of NEAT. The patients/participants provided their written informed consent to participate in this study.

## Author contributions

IA, RU, AJ, and BS initiated and designed the study. AJ, BS, and AK collected the field data and blood samples under the supervision of IA and FZ. AK, MS, and EA performed laboratory analysis. MZ and HN entered the data into Excel. IA, AA, and FZ performed the statistical analysis, while AA and MZ wrote the first draft of the manuscript. IA and FZ supervised the study and the guarantor. All authors contributed to the critical revision and editing of the manuscript. All authors contributed to the article and approved the submitted version.

## Funding

This research was funded by the Researchers Supporting Project No: RSP-2021/45, King Saud University, Riyadh, Saudi Arabia.

## Conflict of interest

The authors declare that the research was conducted in the absence of any commercial or financial relationships that could be construed as a potential conflict of interest.

## Publisher's note

All claims expressed in this article are solely those of the authors and do not necessarily represent those of their affiliated organizations, or those of the publisher, the editors and the reviewers. Any product that may be evaluated in this article, or claim that may be made by its manufacturer, is not guaranteed or endorsed by the publisher.
