# Supplementary material for: Improvement in cardio-metabolic health and immune signatures in old individuals using daily chores (Salat) as an intervention: A randomized crossover study in a little-studied population
Source: Front Public Health. 2022 Oct 24;10:1009055. doi: 10.3389/fpubh.2022.1009055 (PMC9638034; doi:10.3389/fpubh.2022.1009055)
Supplement: Supplementary file 1 [file Data_Sheet_1.docx]

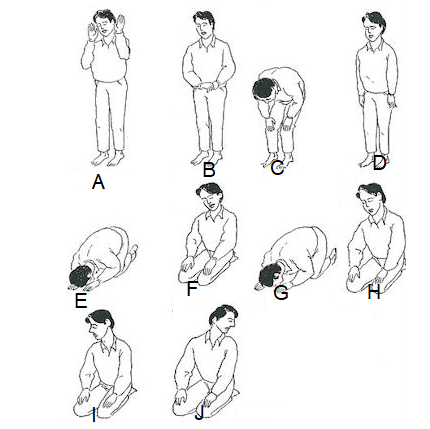


**Figure 1. Various physical postures during *Salat* offering**

*Salat* involves recitations (A) and specific body positions, for example, standing (B,D), bowing (C), prostration (E), sitting (F, H) and turning the head to the right then to the left shoulder (I, J). Salat is initiated with takbir (A), which is the movement of raising hands to the level of one's face so that the thumbs touch the ear lobule. It is then followed by standing (qiyam) for up to 60-90 seconds (B), followed by bowing (rukuk) of 5-10 seconds (C), and then standing again for 2-5 seconds (D). The person then moves from standing to prostration (sujud) for 5-10 seconds (E), followed by sitting (tahiyyat) for 2-5 seconds (F), and then back to prostration 5- 10 seconds (G), followed by sitting (tahiyyat) for 20-30 seconds (H). All of this activity concludes a Rakat, and an act of Salat may consist of 2-4 Rakats. At the end of Salat the person turns the head to both shoulders, first right and then left (I, J). In the comfortable standing position, the center of pressure is usually midway between the feet. In a standing position, an individual will raise his hands up to the ear lobe and bring them down one after another by holding the left wrist with the right hand on the abdomen, above the navel, or on the chest. This helps in standing erect in a right position to avoid undue stress on the backbone. During voluntary clenching of the right hand, blood flow may increase in the hand/limb area of the left motor cortex, and the corresponding sensory areas in the post-central gyrus. The individual offering ‘salat’ utters some Quran versus in Arabic which may also contribute in activating speech muscles of the oral cavity. Bowing after standing and uttering some verses from the Quran is done. Bowing is done by forward movement of the vertebral column, resulting in activation of the back muscles especially the Para spinal muscles and this movement is supported by two straight hands grasping the hyperextended knees. After a few seconds, the individual gradually reverts to his/her normal standing position. Prostration is the substance of ‘Salat’. It is done from the standing position to kneeling, putting the head down and touching the ground with the forehead, with the palms remaining parallel to the ears, and touching the ground with the flexed elbows for a few seconds. After standing and bowing, and proceeding to prostration, sitting is done on the left leg knee flexed with the inverted Dorsi-flexed ankle and flexed right knee and metatarsophalangeal joint for a couple of minutes. After that, Salat is concluded by looking over one’s right and left shoulder wishing peace for mankind.


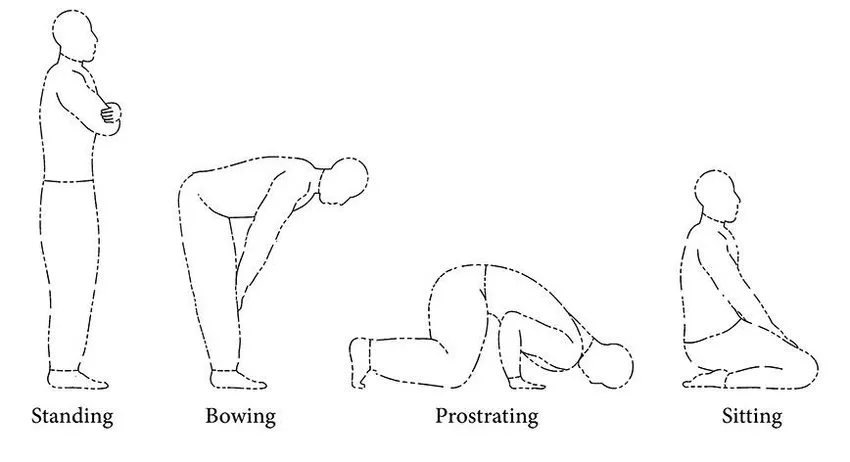


**Figure 2. Side-view of various physical postures during *Salat* offering**

**References**

1. Doufesh H, Ibrahim F, Ismail NA, Ahmad WAW. Assessment of heart rates and blood pressure in different salat positions. J Physical Therapy Sci. 2013; 25:211-4.
2. Alwasiti HH, Aris I, Jantan A. EEG activity in Muslim prayer: a pilot study. Maejo Inter J Sci Tech. 2010; 4:496-511.
3. Yucel S. The effects of prayer on Muslim patients’ well-being (microform). Boston: Boston University School of Theology, 2007
4. . Reza MF, Urakami Y, Mano Y. Evaluation of a new physical exercise taken from salat (prayer) as a short-duration and frequent physical activity in the rehabilitation of geriatric and disabled patients. Annal Saudi med. 2001; 22:177-80.
5. Salahuddin MHR, Abas WW, Osman NA, Ibrahim F, Rahim R. Preliminary Study: The Impact of Moderate Exercises on Biomechanical Response of the Humans Muscles. Biomed. 2008; 21:49-452.
